# Stridor and respiratory failure due to tracheobronchomalacia: case report and review of the literature

**DOI:** 10.1590/S1516-31802012000100011

**Published:** 2012-02-13

**Authors:** Ramon Andrade de Mello, Adriana Magalhães, Abílio José Vilas-Boas

**Affiliations:** I MD. Doctoral Student of Medicine and Molecular Oncology, School of Medicine, University of Porto, Portugal; Resident of Medical Oncology at Francisco Gentil Portuguese Oncology Institute, Porto, Portugal.; II MD. Pulmonology Specialist in the Pulmonology Department, Hospital São João, Porto, Portugal.; III MD. Internal Medicine Specialist and Head of B4 Internal Medicine Unit, Internal Medicine Department, Hospital São João, Porto, Portugal.

**Keywords:** Tracheobronchomalacia, Respiratory sounds, Pulmonary disease, chronic obstructive, Dyspnea, Respiratory insufficiency, Traqueobroncomalácia, Sons respiratórios, Doença pulmonar obstrutiva crônica, Dispnéia, Insuficiência respiratória

## Abstract

**CONTEXT:**

Tracheobronchomalacia (TBM) results from structural and functional abnormalities of the respiratory system. It is characterized by excessive collapse: at least 50% of the cross-sectional area of the trachea and main bronchi. In this paper, we present a rare case of a patient with TBM who first presented with stridor and respiratory failure due to exacerbation of chronic bronchitis.

**CASE REPORT:**

An 81-year-old Caucasian man was admitted presenting coughing, purulent sputum, stridor and respiratory failure. He had a medical history of chronic obstructive pulmonary disease (COPD) and silicosis and was a former smoker. Axial computed tomography on the chest revealed marked collapse of the trachea in its middle third. Bronchoscopy showed characteristics compatible with TBM. He was treated with noninvasive ventilation, without any good response. Subsequently, a Dumon Y stent was placed by means of rigid bronchoscopy. After the procedure, he was discharged with a clinical improvement.

**CONCLUSION:**

TBM is fatal and often underdiagnosed. In COPD patients, stridor and respiratory failure may be helpful signs that should alert physicians to consider TBM as an early diagnosis. Thus, these signs may be important for optimizing the treatment and evolution of such patients.

## RESUMO

**CONTEXTO:**

Traqueobroncomalácia (TBM) é resultado de alterações funcionais e estruturais do aparelho respiratório. É caracterizada pelo colapso excessivo de pelo menos 50% da área de secção transversal da traqueia e dos brônquios principais. Neste trabalho, descrevemos um raro caso de paciente com TBM que primeiro apresentou estridor e insuficiência respiratória devido à exacerbação da bronquite crônica.

**RELATO DE CASO:**

Homem de 81 anos de idade, caucasiano, foi admitido apresentando tosse, expectoração purulenta, estridor e falência respiratória. Ele apresentava história médica prévia de doença pulmonar obstrutiva crônica (DPOC), silicose e era ex-tabagista. A tomografia axial computadorizada de tórax revelou marcado colapso da traqueia em seu terço médio. A broncoscopia mostrou aspectos compatíveis com TBM. Foi submetido a ventilação não invasiva, sem boa resposta. Na sequência, foi colocado *stent* Dumon em Y por broncoscopia rígida. Após o procedimento, o paciente teve alta com melhora clínica.

**CONCLUSÃO:**

TBM é uma entidade fatal e muitas vezes subdiagnosticada. Em pacientes com DPOC, o estridor e a insuficiência respiratória podem ser sinais úteis que devem alertar os médicos a considerar TBM como diagnóstico precoce. Assim, pode ser importante para otimizar o tratamento e a evolução dos pacientes.

## INTRODUCTION

Tracheobronchomalacia (TBM) is a disease of the central airways in which tissue fragility develops in the trachea due to destruction of the supporting cartilage, thereby leading to structural and functional instability of the respiratory system.^1^ TBM is characterized by excessive collapse, consisting of at least 50% of the cross-sectional area of the trachea and the main bronchial lumen, in association with increased compliance of the airway wall, supporting cartilaginous rings, or both.^2^ Histopathological studies on patients with TBM have shown inflammatory infiltrates with T lymphocytes and macrophages.^1^ The incidence of TBM accounts for about 1% of all patients undergoing bronchoscopy.^3^ We present a rare case of a patient with TBM who first presented with stridor and respiratory failure due to exacerbation of bronchitis. We focus on the diagnostic difficulties and present a literature review.

## CASE REPORT

An 81-year-old Caucasian man, who was a former bricklayer, was referred to the emergency department of Hospital São João, a central university hospital, in Porto, Portugal, presenting coughing, purulent sputum, dyspnea and stridor that had lasted for one week. He had a medical history of chronic obstructive pulmonary disease (COPD) with a restrictive and obstructive pattern on functional respiratory tests using spirometry, presenting forced expiratory volume in the first second (FEV1) of 60% and Tiffeneau index of 54%. He was a former smoker (about 25 pack-years), presented silicosis and had been hospitalized due to respiratory infection and insufficiency several times previously over the last six months. He did not have any relevant family history.

**Figure 1.**
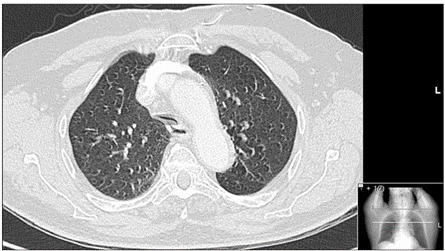
Chest computed tomography showing lung emphysema and cross-sectional collapse of the trachea.

On physical examination, he presented central cyanosis, tachypnea and dispersed crackles. The blood arterial gas analysis showed hypoxia (pH = 7.43, pO_2_ = 56 mmHg, pCO_2_ = 30.9 mmHg, HCO_3_^-^ = 20 mmol/l, SaO_2_ = 90.5%). The other blood analyses revealed leukocytosis of 19.75 x 10^9^/l (normal range: 4.0-11.0 x 10^9^/l), plasma urea of 96 mg/dl (reference value: 10-50) and plasma creatinine of 2.5 mg/dl (reference value: 0.6-1.0). He was started on oxygen and empirical antibiotic therapy: eight days of piperacillin/tazobactam were completed. In order to rule out pulmonary thromboembolism, chest computed tomography was performed. This revealed a marked collapse of the trachea in its middle third, without any intraluminal or extrinsic mass (Figure 1).

**Figure 2.**
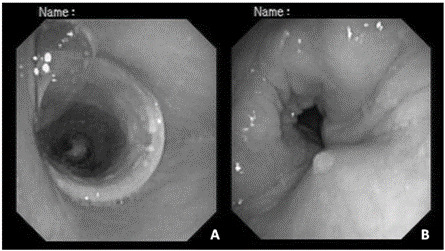
A) Well tolerated tracheal Dumon Y stent without granulation tissue; B) Expiratory airway collapse of left distal extremity by about 30% in relation to original size, but without inspiratory collapse.

The patient was kept in hospital, and noninvasive ventilation with continuous positive airway pressure (CPAP) was introduced. In order to assess the airway collapse, bronchoscopy was performed. This showed a trachea presenting marked expiratory collapse, with contact between the anterior and posterior walls over a length of 2 cm, at the transition from the upper third to the middle third, and extending into both main bronchi. These characteristics were compatible with TBM. The patient was kept on CPAP until a Dumon Y stent was inserted by means of rigid bronchoscopy (Figure 2).

In order to study the TBM etiology, an immunological evaluation was performed, consisting of anti-neutrophil cytoplasmic antibody (ANCA) directed to proteinase 3 (PR3), ANCA myeloperoxidase (MPO), antibody anti-basal membrane, anti-centromere, anti-Jo, anti-RNP (ribonucleoprotein), anti-scleroderma (anti-Slc70), anti-Smith (Anti-Sm), Anti-SSa, Anti-SSb and anti-nuclear antibody. This did not show any abnormalities. The patient did not present any gastroesophageal reflux disease (GERD) or any post-traumatic event. On discharge, he did not present any stridor or dyspnea. His clinical status was good and there was no need for noninvasive ventilation. Although the patient did not undergo any quality-of-life questionnaire or any post-stenting respiratory test reassessment, he mentioned, during the postoperative follow-up, that he felt subjectively that his quality of life was better than it had been at the beginning of this treatment.

## DISCUSSION

To our knowledge, the first case of TBM was described by Czyhlarz et al.^4^ at the end of the 19^th^ century. Some decades later, in 1949, Lemoine et al.^5^ were the first to describe acquired tracheal enlargement in adults, by means of bronchoscopy. In 1952, TBM was described by Holinger et al. as tracheal flaccidity and collapse during the expiratory phase of respiration, based on a bronchoscopy study on three patients presenting with stridor, cyanosis and obstruction.^6^ The clinical symptoms normally include dyspnea, chronic coughing and recurrent respiratory infections.^7^ In the present report, the patient presented stridor that was treated with CPAP.

According to Carden et al.,^8^ TBM is classified into primary (genetic or idiopathic) and secondary, such as post-traumatic, emphysema/COPD, chronic infection/bronchitis, chronic inflammation, extrinsic compression of the trachea (malignancy, benign tumor, cysts, abscesses or aortic aneurism) or vascular rings. TBM has been also correlated with tracheoesophageal fistula and GERD.^6^ In 1992, Mair and Parsons proposed another classification according to etiology: type 1, also called primary TBM, a congenital abnormality due to cartilage immature or abnormal cartilage composition; type 2, extrinsic tracheal compression of otherwise normal tracheal cartilage; and type 3, acquired from severe inflammatory disease, such as relapsing polychondritis (RP),^9^ prolonged increased airway pressures or long-term tracheotomy.^10^

Because of our patient’s clinical context and the results from examinations already carried out, we concluded that TBM was associated with COPD and recurrent respiratory infections. A systematic search in major databases (Table 1) using the description for the main clinical features observed in our patient found some reports that showed the difficulties in dealing with TBM and the therapeutic implications for improving the clinical outcomes from TBM.^11^^,^^12^^,^^13^^,^^14^ One report showed that RP associated with TBM may have a worse prognosis.^14^ Fortunately, our patient did not have the criteria for RP, such as recurrent severe episodes of inflammation of cartilaginous structures of the external ear, nose and peripheral joints,^9^ thus ruling out this entity.

Advances in imaging assessments using computed tomography (CT) and interventional bronchoscopy have improved the recognition of TBM and the clinical approach towards it.^3^ Traditionally, bronchoscopy has been the gold standard examination for diagnosing TBM.^2^ However, recently, the use of paired multidetector inspiratory-dynamic expiratory computed tomography (MDCT) has been shown to be an effective, noninvasive examination for diagnosing TBM.^15^ Conventional CT scan techniques allow radiologists to make a diagnosis of fixed-airway narrowing, but only MDCT enables a diagnosis of TBM, through demonstrating excessive expiratory collapse of the airways throughout the respiratory cycle.^16^ In the present case, the chest CT initially suggested airway collapse, probably because the patient was not collaborative during the scan inspiration, and an expiratory image was obtained instead. The bronchoscopy was thus decisive for the diagnosis and for addressing the respiratory failure and stridor when the Dumon Y stent was emplaced. 

There is no consensus regarding treatments for TBM.^1^ Identifying patients with symptoms and severe disease is still a challenge. The possible approaches are pharmacological treatment, such as treatments for COPD and GERD, noninvasive ventilation using CPAP, stents (silicon, metal or hybrid) and surgery, such as tracheobronchoplasty, aortopexy and tracheopexy.^1^ Furthermore, since MDCT techniques provide three-dimensional preoperative imaging assessments, they can improve surgeons’ approaches by facilitating their understanding of patients’ anatomical issues.^17^ However, Majid et al. reported in a prospective study that selected patients with severe diffuse TBM associated with COPD and asthma could benefit highly from tracheobronchoplasty with posterior tracheobronchial splitting using a Marlex mesh, thereby reestablishing the normal airway anatomy and preventing expiratory collapse while maintaining normal mucociliary function.^18^ Nevertheless, they also concluded that patients with chronic lung disease who presented symptom improvement after stent use should be considered for a definitive surgical repair as soon as it becomes possible to tolerate this procedure.

**Table 1.** Systematic search strategy used by the authors on December 27, 2010

Database	Search strategy	Results	
PubMed	“Tracheobronchomalacia” (Mesh)AND “respiratory sounds” (Mesh) AND “Respiratory failure” (Mesh)	1 manuscript	1 case report^11^
	“Tracheobronchomalacia”(Mesh) AND “respiratory sounds” (Mesh)	5 manuscripts	4 case reports 1 original article^12^
	“Tracheobronchomalacia”(Mesh) AND “Respiratory insufficiency”(Mesh)	9 manuscripts	2 reviews 4 case reports 3 original articles
Cochrane	“Tracheomalacia”	1 manuscript	1 review^13^
Embase	“Tracheomalacia” AND “breath sounds”	11 manuscripts	5 case reports 2 case series 2 original articles 2 reviews
Scirus	“Tracheomalacia” AND “breath sounds”	2 manuscripts	2 reviews
Lilacs	“Tracheobronchomalacia” (DeCS)	5 manuscripts	1 case report^14^ 4 original articles
SciELO	“Tracheobronchomalacia” (DeCS)	6 manuscripts	6 original articles

MeSH = Medical Subject Heading; DeCS = Descritores em Ciências da Saúde; Lilacs = Literatura Latino-Americana e do Caribe em Ciências da Saúde; SciELO = Scientific Electronic Library Online.

## CONCLUSION

TBM is a rare and sometimes underdiagnosed disease.^3^ Symptomatic obstruction of tracheal long-segment or bronchial portions of the airways may be related to life-threatening dysfunction of the respiratory system.^2^ In COPD patients, stridor and dyspnea due to acute respiratory failure might be considered to be important signs relating to TBM. Thus, such situations should alert physicians towards suspecting this entity and lead to improved diagnostic investigation. A prompt and correct approach is very important for optimizing the treatment and outcome.
